# Deficiency of Mettl3 in Bladder Cancer Stem Cells Inhibits Bladder Cancer Progression and Angiogenesis

**DOI:** 10.3389/fcell.2021.627706

**Published:** 2021-02-18

**Authors:** Ganping Wang, Yarong Dai, Kang Li, Maosheng Cheng, Gan Xiong, Xiaochen Wang, Shuang Chen, Zhi Chen, Jianwen Chen, Xiuyun Xu, Rong-song Ling, Liang Peng, Demeng Chen

**Affiliations:** ^1^Center for Translational Medicine, Institute of Precision Medicine, The First Affiliated Hospital, Sun Yat-sen University, Guangzhou, China; ^2^Institute for Advanced Study, Shenzhen University, Shenzhen, China; ^3^Department of Oral and Maxillofacial Surgery, Hospital of Stomatology, Guanghua School of Stomatology, Sun Yat-sen University, Guangzhou, China; ^4^Department of Oncology, Chinese PLA General Hospital, Beijing, China

**Keywords:** Mettl3, cancer stem cell, bladder cancer, m^6^A, angiogenesis

## Abstract

RNA N6-methyladenosine is a key step of posttranscriptional modulation that is involved in governing gene expression. The m^6^A modification catalyzed by Mettl3 has been widely recognized as a critical epigenetic regulation process for tumorigenic properties in various cancer cell lines, including bladder cancer. However, the *in vivo* function of Mettl3 in bladder cancer remains largely unknown. In our study, we found that ablation of Mettl3 in bladder urothelial attenuates the oncogenesis and tumor angiogenesis of bladder cancer using transgenic mouse model. In addition, conditional knockout of Mettl3 in K14^+^ bladder cancer stem cell population leads to inhibition of bladder cancer progression. Coupled with the global transcriptome sequencing and methylated RNA immunoprecipitation sequencing results, we showed that deletion of Mettl3 leads to the suppression of tyrosine kinase endothelial (TEK) and vascular endothelial growth factor A (VEGF-A) through reduced abundance of m^6^A peaks on a specific region. In addition, the depletion of Mettl3 results in the decrease in both messenger RNA (mRNA) and protein levels of TEK and VEGF-A *in vitro*. Taken together, Mettl3-mediated m^6^A modification is required for the activation of TEK–VEGF-A-mediated tumor progression and angiogenesis. Our findings may provide theoretical basis for bladder cancer treatment targeting Mettl3.

## Introduction

Bladder cancer (BCa) is one of the most common malignancies worldwide, with approximately 200,000 death and 550,000 new cases yearly (Bray et al., 2018). Patients with primary tumor could be treated by surgical resection, but it is limited by the several major progressions like metastasis and recurrence (Griffiths and Action on Bladder Cancer, 2013; Crabb and Douglas, 2018). Furthermore, advanced BCa is characterized by high recurrence rate and poor prognosis, making it a growing social and medical challenge. During the past decades, despite great efforts on larger range of novel therapies for advanced BCa, limited clinical efficacy is obtained (Powles et al., 2017; Powles and Morrison, 2018). Therefore, a deeper understanding of specific mechanism in BCa metastasis and recurrence as well as the exploration of effective targets for better disease control has become major goals to be attained.

N6-methyladenosine (m^6^A), a predominantly internal modification of RNA in mammalian cells, is demonstrated to regulate RNA translation, degradation, splicing, export, and folding (Wang et al., 2014, 2015; Zhao et al., 2014; He et al., 2019). m^6^A regulation is dynamic and reversible epigenetic modification mediated by three components: “writer” complex, “eraser,” and “reader” (Jia et al., 2011; Zheng et al., 2013; Liu et al., 2014; Li et al., 2017). Methyltransferase-like 3 and 14 (Mettl3 and Mettl14) and their cofactors, Wilms tumor 1 associated protein (WTAP), VIRMA (KIAA1429), and RBM15 function as writers and form m^6^A methyltransferase complex (MTC), which catalyzes A (adenosine) to become m^6^A. Emerging insights into the role of m^6^A regulation in biological processes and pathogenesis of diseases has been uncovered in recent years, mainly including cell fate determination, embryonic development, and tumor progression (Roundtree et al., 2017; Frye et al., 2018). For example, data show that m^6^A modification pathway could exert carcinogenic or anticancer influence in various tumors for the expression pattern of the same m^6^A-regulated proteins, and their target messenger RNAs (mRNAs) may differ with the types of tumor (Wang et al., 2018; Sun et al., 2019). Previous studies have demonstrated that Mettl3-mediated m^6^A modification directly regulates AFF4/NF-κB/MYC signaling network and ITGA6 mRNA, hence promoting BCa progression (Cheng et al., 2019; Jin et al., 2019). However, the understanding of the molecular mechanism of m^6^A in BCa *in vivo* remains largely undefined.

N-Butyl-N-(4-hydroxybutyl) nitrosamine (BBN) is a well-known carcinogen that can induce mouse bladder cancer, which resembles human bladder cancer (Kelsey, 2018). In addition, *Upk3a*^*creER*^ transgenic mouse expresses a tamoxifen-inducible Cre recombinase in bladder urothelium and has been widely used for *in vivo* study of BCa (Korac-Prlic et al., 2020). Emerging evidence have shown that cancer stem cells can be used as a predictor for overall patient survival and process properties that are correlated with tumor progression, therapy resistance, and recurrence (Kreso and Dick, 2014). Cancer stem cells (CSCs) in BCa are responsible for unresponsive chemotherapy, and this unexpected consequence can be abrogated by celecoxib (Kurtova et al., 2015). Recently, an increased level of K5/6 and K14 has been found in basal-like BCa (Zhao et al., 2019). K14^+^ bladder cancer cells play an important role in urothelial homeostasis and are described as a marker for the BCa stem cell population, which facilitates tumorigenesis and chemotherapy resistance *in vivo* (Volkmer et al., 2012; Kurtova et al., 2015; Papafotiou et al., 2016).

Once solid tumor grows up to a diameter of 2 mm, vascular system is a necessity for continued growth. Then, angiogenesis is acquired for tumor cells and function as a selective way to push the limits. Moreover, for tumor growth and invasion, angiogenesis has been considered to be the basic pathological feature during the process. Tyrosine kinase endothelial (TEK), also called TIE2, is an effective target for malignancy treatment by inhibition of tumor angiogenesis through ANG2/TIE2 axis (Mazzieri et al., 2011). Vascular endothelial growth factor A (VEGF-A) is a well-known growth factor that activates angiogenesis to promote cell migration, therefore collaborating with tumorigenesis (Goel and Mercurio, 2013). As part of tumor comprehensive therapy, antiangiogenesis therapy has been widely used in clinical practice. Furthermore, markers of angiogenesis such as CD31, CD34, and CD105 could serve as an independent prognostic factor in cancers (Tanaka et al., 2001; Randall et al., 2009). Thus, understanding the specific pathway of regulating angiogenesis in BCa is essential for developing future diagnostic and therapeutic strategies.

In this study, we aim to identify the role that m^6^A promotes BCa progression via focusing on the key methyltransferase Mettl3. Conditional knockout Mettl3 among K14^+^ or Upk3a^+^ cells *in vivo* is found to restrain BCa development through disabling angiogenesis, which is further confirmed by RNA sequencing and analyzing the expression pattern of angiogenesis key factors. Collectively, we first verify the function of m^6^A mediated by Mettl3 for BCa and expatiate on the network of Mettl3 in governing tumor angiogenesis *in vivo*.

## Materials and Methods

### Animals

The transgenic mice were maintained under specific pathogen-free conditions at the Center of Experimental Animal of Sun Yat-sen University. All procedures and use of animal experiments were approved by Sun Yat-sen University Animal Care and Use Committee.

*Upk3a*^*CreER*^ (JAX stock: 015855), *K14*^*CreER*^ (JAX stock: 005107), and *C57BL/6J* background mice were obtained from the Jackson Laboratory. *Mettl3*^*flox/flox*^ and *Mettl3*^*KI*^ mice were obtained as previously described (Liu et al., 2020). For induction of BCa, 6–8-week-old mice were treated with drinking water containing 500 μg/ml BBN for 16 weeks and then given normal water for another 10 weeks. Tamoxifen was intraperitonelly injected to the mice with 0.08 mg/g of body weight each day for 3 days in order to inductively knock out the target gene. Then, the experimental mice were sacrificed for collection of BCa samples.

### Cell Culture and Transfection

Bladder cancer cell lines of T24 and UMUC-3 were cultured in Roswell Park Memorial Institute (RPMI)-1640 (Gibco, United States) medium supplemented with 10% fetal bovine serum (Gibco, United States) and in Dulbecco’s modified Eagle’s medium (DMEM) basic (Gibco, United States) medium supplemented with 10% fetal bovine serum (FBS), respectively. T24 and UMUC-3 cells were grown in the cell culture incubator with 5% CO_2_ at 37°C. Stable transfection of plasmids was performed using Lipofectamine 2000 (Invitrogen, United States) according to the manufacturer’s instructions. The following short hair RNA (shRNA) sequences were applied to knock down Mettl3 in this study: shMettl3-1, 5′-CCGGGCTGCACTTCAGACGAATT-**CTCAAGAGA-**AATTCGTCTGAAGTGCAGCTTTTTTG-3′; shMettl3-2, 5′-CCGGGCAAGAATICTGTGACTAT-**CCTCAAGAGA**-ATAGTCACAGAATTCTTGCTTTTTTG-3′; shMettl3-3, 5′-CCGGGCTCAACATACCCGTACTA-**CCTCAAGAGA**-TAGTACGGGTATGTTGAGCTTTTTTG-3′; control, 5′-CCGGTTCTCCGAACGTGTCACGT-**TTCAAGAGA-**ACGTTGACACGTTCGGAGAATTTTTTG-3′.

### Immumohistochemical Staining and IHC Scoring

All the BCa specimens collected were embedded in paraffin and then cut into 5-μm thick sections. The samples were deparaffinized for 10 min in two different tanks with Histo-clear fluid after heating at 65°C for 30 min; then, these sections were hydrated in different gradients of ethanol. The samples were incubated for 10 min in 3% H_2_O_2_ to block endogenous peroxidases. After blocking, antigen retrieval was performed with ethylenediaminetetraacetic acid (EDTA) buffer (Beyotime Biotechnology, Shanghai, China) in a microwave oven with medium power for 5 min and high power for another 5 min (avoid complete drying of sections). Specific primary antibody (1:200) was used at 4°C overnight in a wet box. Then, antiprimary secondary antibody was applied and incubated for 30 min at room temperature. The secondary antibody was removed and 3,3′-diaminobenzidine (DAB) dripped upon sections, and the color development index was observed. Hematoxylin was conducted for counterstaining cell nucleus. The immunohistochemistry (IHC) score was calculated by the product of staining intensity and frequency. The intensity was defined as 0 (negative), 1 + (weakly positive), 2 + (positive), or 3 + (strongly positive), and the frequency was considered to be percentage of positive cells.

### H&E Staining

The sections were prepared by the same method as described above and stained with hematoxylin for 5 min. Next, hydrochloric acid alcohol was utilized to separate color for 5 s, and the sections were further stained with eosin for 2 min. After that, all samples were dehydrated by gradient alcohol and sealed with neutral balsam medium.

### Immunofluorescence Staining

The BCa specimens were fixed in 4% paraformaldehyde and permeabilized with 0.1% Triton X-100 (Sigma, United States), followed by blocking with 1% bovine serum in phosphate-buffered saline (PBS) at room temperature. The sections were stained with primary antibody at 1:100 and were incubated at 4°C overnight, followed by application of the corresponding secondary antibody conjugated with DyLight 488 and Fluor 594 (Invitrogen, United States). At the same time, normal serum was used as a control to guarantee the specificity of the primary antibody. The nuclei were counterstained with 4′,6-diamidino-2-phenylindole (DAPI) at 1:1000 for 1 min. Images were captured with an upright fluorescence microscope (Nikon, Japan).

### RNA-Sequencing

Oligo (dT)-attached magnetic beads were used to purified mRNA from BBN-induced *K14^*CreER*^; Mettl3^*flox/flox*^* mice or their control littermates carrying bladder tumors. Purified mRNA was fragmented into small pieces with fragment buffer at appropriate temperature. Then, first-strand complementary DNA (cDNA) was generated using random hexamer-primed reverse transcription, followed by a second-strand cDNA synthesis. Afterward, A-Tailing Mix and RNA Index Adapters were added by incubating to end repair. The cDNA fragments obtained from the previous step were amplified by PCR, and products were purified by Ampure XP Beads and then dissolved in EB solution. The product was validated on the Agilent Technologies 2100 bioanalyzer for quality control. The double-stranded PCR products from the previous step were heated denatured and circularized by the splint oligo sequence to get the final library. The single-strand circle DNA (ssCir DNA) was formatted as the final library. The final library was amplified with phi29 to make DNA nanoball (DNB), which had more than 300 copies of one molecular; DNBs were loaded into the patterned nanoarray, and single-end 50 bases reads were generated on BGIseq500 platform (BGI-Shenzhen, China).

### RNA Extraction and RT-qPCR

The bladder tissues were collected from BBN-induced mice carrying bladder primary tumor. Frozen tissues by liquid nitrogen were dissociated with 1 ml TRIzol Reagent (Invitrogen, United States) per 50 mg of tissue in Tissue Lyser. After dissociation, 0.2 ml of chloroform per 1 ml of TRIzol Reagent was added and incubated for 3 min. Then, samples were centrifuged for 15 min at 12,000 × *g* at 4°C, and the upper aqueous phase containing total RNA was collected. The supernatant was added equivalent isopropyl alcohol, gently blended, and placed for 10 min at room temperature, followed by a centrifugation again, and the supernatant was discarded. One milliliter of 75% ethanol diluted with diethylpyrocarbonate (DEPC) water was added to each sample, and the supernatant was discarded after the centrifugation (4°C, 12,000 rpm and 5 min). Moderate amount of DEPC-treated water was added to the precipitation after evaporation of ethanol. The 500 ng of RNA from each sample was reverse-transcribed with the reverse-transcription kit (Takara Biotechnology Co., Ltd., Dalian, China). PCR reaction conditions were as follows: 98°C for 30 s, followed by 32 cycles at 95°C for 30 s, 55°C for 30 s, and 72°C for 30 s. The specific primers for target genes of mice in quantitative PCR (qPCR) were as follows: TEK (forward), 5′- GAACTGAGGACGCTTCCACATTC- 3′, TEK (reverse), 5′- TCAGAAACGCCAACAGCACGGT- 3′; VEGF-A (forward), 5′- CTGCTGTAACGATGAAGCCCTG-3′, VEGF-A (reverse), 5′- GCTGTAGGAAGCTCATCTCTCC-3′; glyceraldehyde 3-phosphate dehydrogenase (GAPDH) (forward), 5′- AGGTCGGTGTGAACGGATTTG-3′, GAPDH (reverse), 5′- GGGGTCGTTGATGGCAACA- 3′. The designed primers for the target genes of BCa cell lines were as follows: TEK (forward), 5′- TTAGCCAGCTTAGTTCTCTGTGG- 3′, TEK (reverse), 5′- AGCATCAGATACAAGAGGTAGGG- 3′; VEGF-A (forward), 5′- AGGGCAGAATCATCACGAAGT-3′, VEGF-A (reverse), 5′- AGGGTCTCGATTGGATGGCA- 3′; β-actin (forward), 5′- CATGTACGTTGCTATCCAGGC-3′, β-actin (reverse), 5′- CTCCTTAATGTCACGCACGAT-3′.

### Western Blot Analysis

The cell culture medium was discarded, and the cells were rinsed with 4°C precooled phosphate-buffered saline (PBS) buffer. Total cell protein lysates were obtained from T24 and UMUC-3 cells after lysing in radioimmunoprecipitation assay (RIPA) (Beyotime Biotechnology, China) for 30 min. Quantified with a BCA assay kit (Cwbiotech, Beijing, China), the samples were mixed with 5 × loading buffer and conducted thermal denaturation at 95°C for 5 min. Then, the protein lysates were subjected to sodium dodecyl sulfate–polyacrylamide gel electrophoresis (SDS-PAGE) and electrotransferred to the polyvinylidene fluoride (PVDF) membrane with a constant current of 300 mA for 1.5 h (Millipore, Germany). Then, 5% skimmed milk was used for blocking, followed by incubation with primary antibody (1:1000) overnight. Tris-buffered saline with Tween 20 (TBST)-diluted secondary antibody was applied and incubated at room temperature for 1 h. The blots were visualized using the enhanced chemiluminescence (ECL) method with Tanon 5200 Multi intelligent imaging system.

### m^6^A-Sequencing

For m^6^A-seq, total RNA was isolated from the bladder tissue with or without Mettl3 knockout using TRIzol reagent. mRNA was further purified from prepared total RNA with the use of the Dynabeads mRNA DIRECT kit (Thermo Fisher) according to the manufacturer’s instructions. The purified RNA was used to interact with anti-m^6^A or control rabbit immunoglobulin G (IgG) antibodies, and the antibody-conjugated samples were incubated with IP buffer-washed Protein G Dynabeads at 4°C overnight. Next day, the isolated products were applied for constructing libraries of inputs (control IgG) and methylated RNA immunoprecipitation sequencing (meRIPs) (anti-m^6^A antibody) with TruSeq Stranded mRNA Sample Prep Kits (Illumina RS-122-2101). Libraries were multiplexed on an Illumina HiSeq 2000 platform. Mapping of reads, calling of m6A peaks, recognition of specific motifs, and further analysis were carried out based on previous study (Dominissini et al., 2013).

### KEGG and Gene Ontology Analysis

To explore the link between Mettl3 and the underlying mechanisms in BCa, the Kyoto Encyclopedia of Genes and Genomes (KEGG) and Gene Ontology Analysis were conducted using ToppGene Suite^1^ and KEGG PATHWAY Database^2^ to analyze the differentially expressed genes (DEGs). The statistical significance of difference was evaluated by *P* value, and *P* < 0.05 was considered statistically significant.

### Gene Set Enrichment Analysis

Gene Set Enrichment Analysis (GSEA) software downloaded from the GSEA website^3^ was performed to determine signaling pathways that were correlated between the Mettl3 knockout and control groups. The annotated gene sets of c5.go.v7.2.symbols.gmt in the Molecular Signatures Database (MSigDB) were selected in GSEA version 4.0. One thousand times of permutations were performed. The normalized enrichment score (NES) was served as the GSEA main statistic. Gene sets with nominal *P* < 0.05, false discovery rate (FDR) < 0.25, and normalized enrichment scores (NES) > 1.0 were considered significantly enriched.

### Statistical Analysis

All the data were analyzed and presented as mean ± SD. Significant tests were conducted with unpaired parametric two-tailed Student’s *t* test for difference analysis between two groups or chi-square for tumor grade analysis. *P* < 0.05 was defined as statistical significance (^∗^*P* < 0.05, ^∗∗^*P* < 0.01, ^∗∗∗^*P* < 0.001, and ^****^P < 0.0001). Data management and statistical analyses were performed using GraphPad Prism software, version 7.04.

## Results

### Deletion of Mettl3 in Urothelium Diminishes the Development of Mouse BCa

**FIGURE 1:**
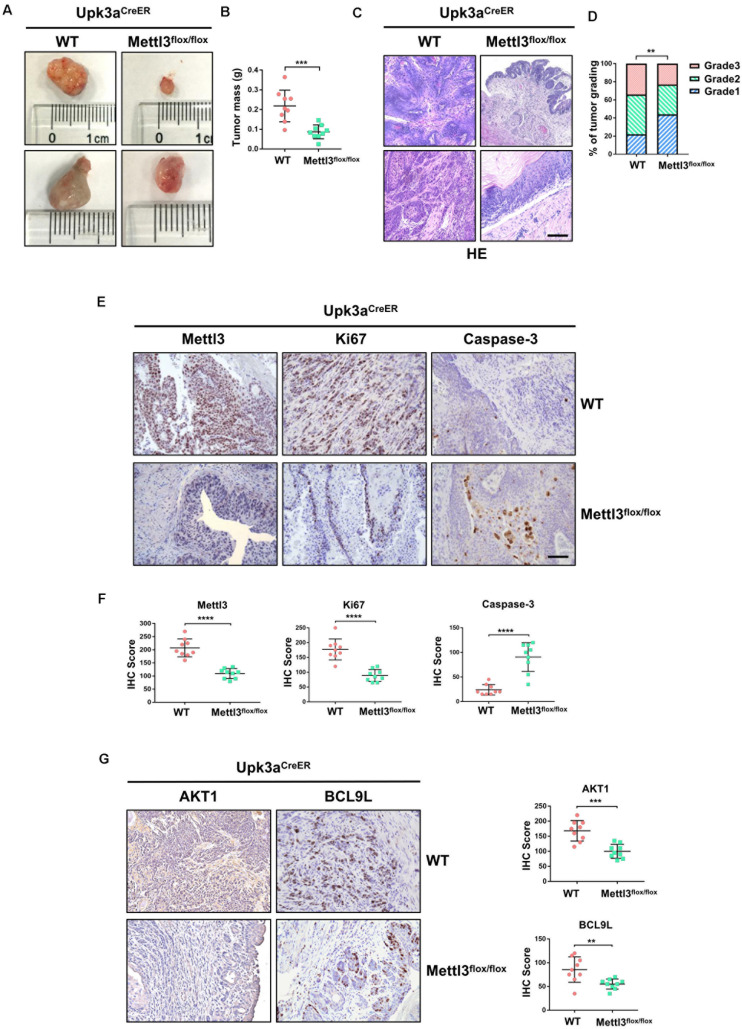
Knockout of Mettl3 in urothelium inhibits the development of bladder cancer (BCa). **(A)** Representative images of BCa lesions from *Upk3a*^*CreER*^, *Mettl3*^*wt/wt*^ or *Upk3a*^*CreER*^, *Mettl3*^*flox/flox*^ mice 26 weeks after BBN treatment of at least three independent groups. **(B)** Statistics of bladder weight of mice in two groups. **(C)** Representative image of H&E staining of BCa section in wild-type and *Mettl3*^*flox/flox*^ mice. Tamoxifen was intraperitoneal injected into N-butyl-N-(4-hydroxybutyl) nitrosamine (BBN)-induced *Upk3a*^*CreER*^ wild-type and *Mettl3*^*flox/flox*^ mice. Bladders were harvested after 24 h tamoxifen treatment. Scale bar, 100 μm. **(D)** Statistics of tumor grading result was based on the H&E staining experiment. **(E)** Immumohistochemical staining for the expression pattern of Upk3a, Mettl3, Ki67, and Caspase-3 in both wild-type and *Mettl3*^*flox/flox*^ mice. Scale bar, 100 μm. **(F)** Immunohistochemical staining scores of Mettl3, Ki67, and Caspase-3 were counted, respectively. **(G)** Immunostaining and IHC score of AKT1 and BCL9L in Upk3aCreER wild type and Upk3aCreER KO mice respectively.

To explore the role of Mettl3 in BCa initiation and development, we first crossed *Upk3a*^*CreER*^ mice with *Mettl3*^*flox/flox*^ mice to generate *Upk3a*^*CreER*^ mice; *Mettl3*^*flox/flox*^ mice that allow us to conditional ablate the expression of Mettl3 in umbrella, intermediate, and basal cells of mouse bladder (Johnson et al., 2016). *Upk3a*^*CreER*^ mice; *Mettl3*^*flox/flox*^ and their control littermates were treated with BBN in their drinking water for 16 weeks before feeding normal water for another 10 weeks, as described previously (Irving et al., 1984). Then, mice were sacrificed for the collection of bladders. Measurement of bladder tumor indicated significantly decreased volume in *Upk3a*^*CreER*^; *Mettl3*^*flox/flox*^ mice compared with the wild-type group (Figure 1A). We further assessed the effect of Mettl3 conditional knockout on tumor weight *in vivo* and found a marked tendency to reduce in tumor weight (Figure 1B). Observation from the H&E staining specimens demonstrated that mice developed lesions on the urothelium of the bladder upon the treatment with BBN, while *Upk3a*^*CreER*^; *Mettl3*^*flox/flox*^ mice carried tumors that were less malignant than *Upk3a*^*CreER*^; *Mettl3*^*wt/wt*^ mice (Figure 1C). The result of BCa grading indicated a higher likelihood to develop into a high-grade tumor in wild-type mice (Figure 1D). IHC staining was applied to examine the protein levels of Mettl3, Ki67, and Caspase-3 protein in BCa (Figure 1E). The reduced expression of Mettl3 and Ki67 with the increased level of Caspase-3 was detected by randomized double-blind IHC scoring in *Upk3a^*CreER*^; Mettl3^*flox/flox*^* mice, manifesting the role of Mettl3 in promoting proliferation and inhibiting apoptosis *in vivo* (Figure 1F). A significant reduction in AKT1 (a proliferation-related factor) (Wang et al., 2019) and BCL9L (an inhibitor of apoptosis) (Huge et al., 2020) was observed by the loss of Mettl3 in Upk3a^+^ BCa cells (Figure 1G). These results indicate that Mettl3 is essential for cellular proliferation and survival in BBN-induced BCa.

### Ablation of Mettl3 in K14^+^ Cancer Stem Cells Inhibits Tumorigenicity and Progression of BCa

**FIGURE 2:**
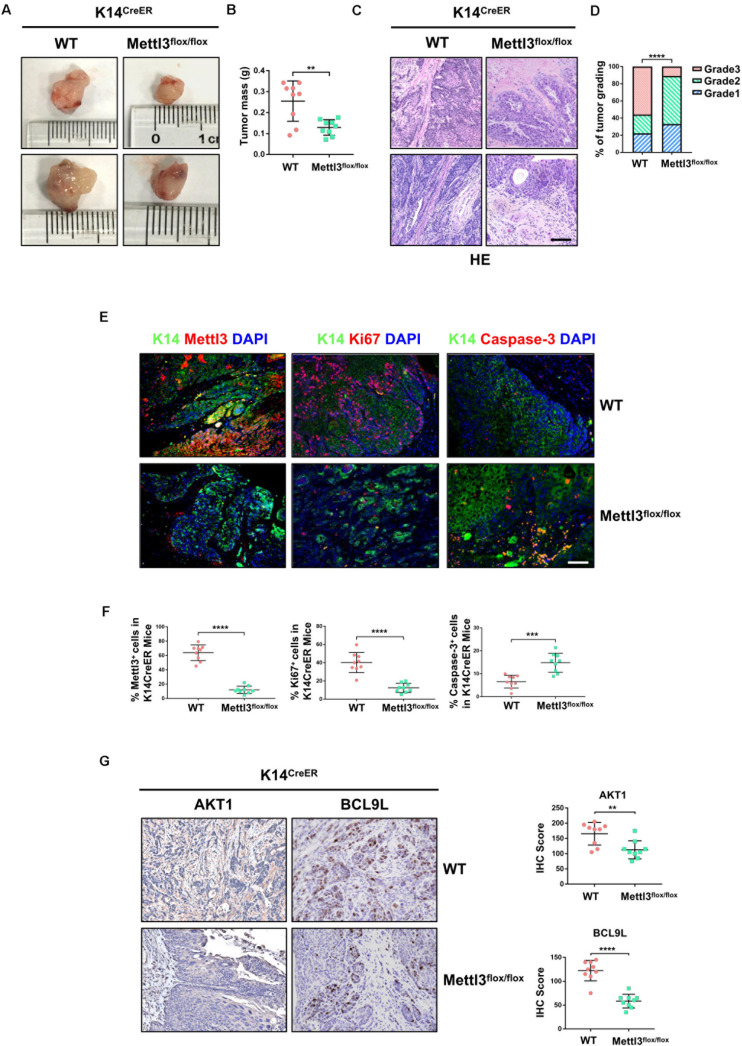
Deletion of Mettl3 in K14^+^ cells displays reduced tumorigenicity, cell proliferation, and survival. **(A)** Representative images of bladder cancer (BCa) lesions from *K14*^*CreER*^, *Mettl3*^*wt/wt*^ or *K14*^*CreER*^, *Mettl3*^*flox/flox*^ mice. **(B)** The bladder weight was calculated in wild-type and *Mettl3*^*flox/flox*^ mice, respectively. **(C)** Representative H&E staining images of BCa section in wild-type and *Mettl3*^*flox/flox*^ mice show the histological atypia. Scale bars, 50 μm. Scale bar, 100 μm. **(D)** Assessment of malignancy in wild-type and *Mettl3*^*flox/flox*^ mice by tumor grading. **(E)** Immunofluorescent staining was used to evaluate the expression of K14, Mettl3 (left), Ki67 (middle), and Caspase-3 (right). 4′,6-Diamidino-2-phenylindole (DAPI) staining was used to mark nucleus. Scale bar, 100 μm. **(F)** Calculation of Mettl3 (left), Ki67 (middle), and Caspase-3 (right) positive cells in wild-type and *K14*^*CreER*^, *Mettl3*^*flox/flox*^ mice. **(G)** Immunostaining and IHC score of AKT1 and BCL9L in K14CreER wild type and K14CreER KO mice respectively.

We next investigated the function of Mettl3 in K14**^+^** CSCs by generation of BBN-induced *K14*^*CreER*^, *Mettl3*^*flox/flox*^ and *K14*^*CreER*^, *Mettl3*^*wt/wt*^ mice. Bladder tumor size was obviously decreased after inducible knockout of Mettl3 in K14**^+^** CSCs (Figure 2A). Similar to the results above, a notably reduced tumor weight was observed with the knockout of Mettl3 (Figure 2B). Then, BCa sections taken from transgenic mice were stained with H&E kit to show the tumor histological morphology. The atypia of cells and tissues was more remarkable in wild-type mice compared with *K14*^*CreER*^, *Mettl3*^*flox/flox*^ mice, revealing that ablation of Mettl3 in K14**^+^** CSCs suppressed the malignant transformation of BCa (Figure 2C). Additionally, bladder tumor grading described Mettl3 as a driving factor of cancer progression (Figure 2D). We then performed immunofluorescence staining to detect the expression of K14 and expression level of Mettl3, Ki67, and Caspase-3 in our transgenic mice model. The data showed that Mettl3 was mostly knocked out after treatment with tamoxifen in K14-derived BCa cells followed by the suppression of Ki67 and enhancement of apoptosis, proving the effect of Mettl3 on elevating cell proliferative potential and limiting apoptosis (Figures 2E,F). The expression of genes associated with proliferation and survival as AKT1 and BCL9L were evaluated by IHC staining in specimens. Yet, the deficiency of Mettl3 led to the decrease in AKT1, while it increased the level of BCL9L (Figure 2G). These data demonstrate that conditional knockout of Mettl3 in K14^+^ CSCs reduced the tumor aggressiveness in mice with BBN induction.

### Overexpression of Mettl3 in Urothelium or K14^+^ CSCs BCa Cells Promotes the Growth of BCa

**FIGURE 3:**
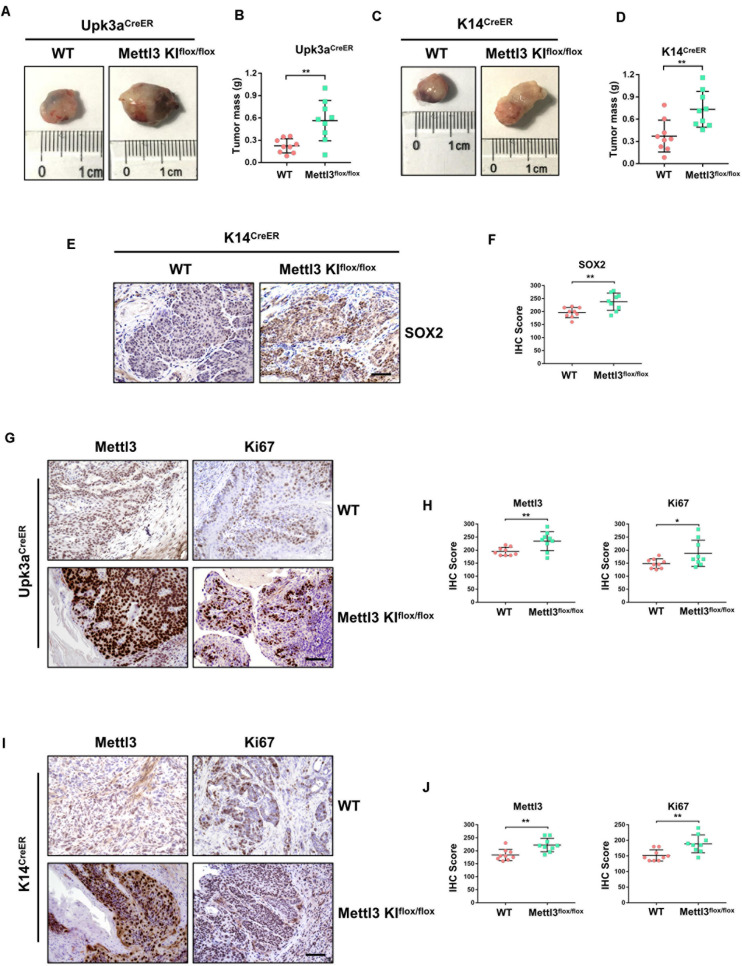
Overexpression of Mettl3 in urothelium or K14^+^ cells promotes bladder cancer (BCa) progression *in vivo*. **(A)** Representative images of BCa in *Upk3a*^*CreER*^ WT (left) and *Upk3a*^*CreER*^ Mettl3 KI (right) mice showing the tumor volume. **(B)** Measurement of tumor mass in Upk3a-specific WT and Mettl3 KI groups, respectively. **(C)** Tumor volume was shown in K14-specific WT (left) and KI (right) mice. **(D)** The weight of tumor collected from *K14*^*CreER*^ wild-type and *K14*^*CreER*^ Mettl3 KI mice was calculated, respectively. **(E)** immunohistochemistry (IHC) staining showing SOX2 expression in WT and Mettl3 KI^*flox/flox*^ mice. Scale bars, 100 μm. **(F)** The IHC score was computed according to the intensity and frequency of SOX2 expression. **(G)** Immunostaining was applied to explore the expression level of Mettl3 and Ki67 in *Upk3a*^*CreER*^ WT or Mettl3 KI mice. Scale bars 100 μm. **(H)** IHC scoring was calculated based on the statistics of staining intensity and ratio of positive cells above. **(I)** Representative IHC staining images of Mettl3 or Ki67 in *K14*^*CreER*^ wild-type and *K14*^*CreER*^ Mettl3 KI mice. Scale bars, 100 μm. **(J)** Calculation of IHC scores according to the immunostaining images above. *p < 0.05; **p < 0.01.

Complementarily, we constructed mice models with inducible conditional overexpression of Mettl3 (Mettl3^*KI*^) in the urothelium or K14^+^ CSCs using *Upk3a*^*CreER*^ or *K14*^*CreER*^. Comparing tumor bulk in *Upk3a*^*CreER*^ wild-type and KI mice, the mice bearing BCa with overexpressed Mettl3 in Upk3a-derived cells developed tumors more aggressively (Figure 3A). It was also found that excessive Mettl3 in cells of Upk3a origin contributed to promoting tumor growth by measuring tumor mass in mice (Figure 3B). Likewise, overexpression of Mettl3 in K14^+^ CSCs cells accelerated tumor growth and progression (Figures 3C,D). We then assessed the level of SOX2, the BCa stem cell marker, in *K14*^*CreER*^ wild type and Mettl3 KI mice, respectively, and a significantly elevated expression of SOX2 was observed after overexpressing Mettl3 (Figures 3E,F) (Zhu et al., 2017). IHC was performed to confirm the knock-in efficiency of Mettl3 (left), and there was an increased Ki67-positive cell along with enhanced staining intensity (right) after overexpression of Mettl3 (Figure 3G). The level of Mettl3 and Ki67 was found to be elevated in *Upk3a*^*CreER*^ KI mice by estimating IHC score (Figure 3F). Subsequently, Ki67 was also discovered to be upregulated in *K14*^*CreER*^, Mettl3 knock-in mice (Figures 3I,J). Taken together, our data demonstrate that overexpressed Mettl3 in bladder urothelium cells or K14^+^ CSCs escalate the tumor aggressiveness in mouse BCa model.

### Mettl3 Regulates the PI3K/AKT Pathway and Tumor Angiogenesis in BCa

**FIGURE 4:**
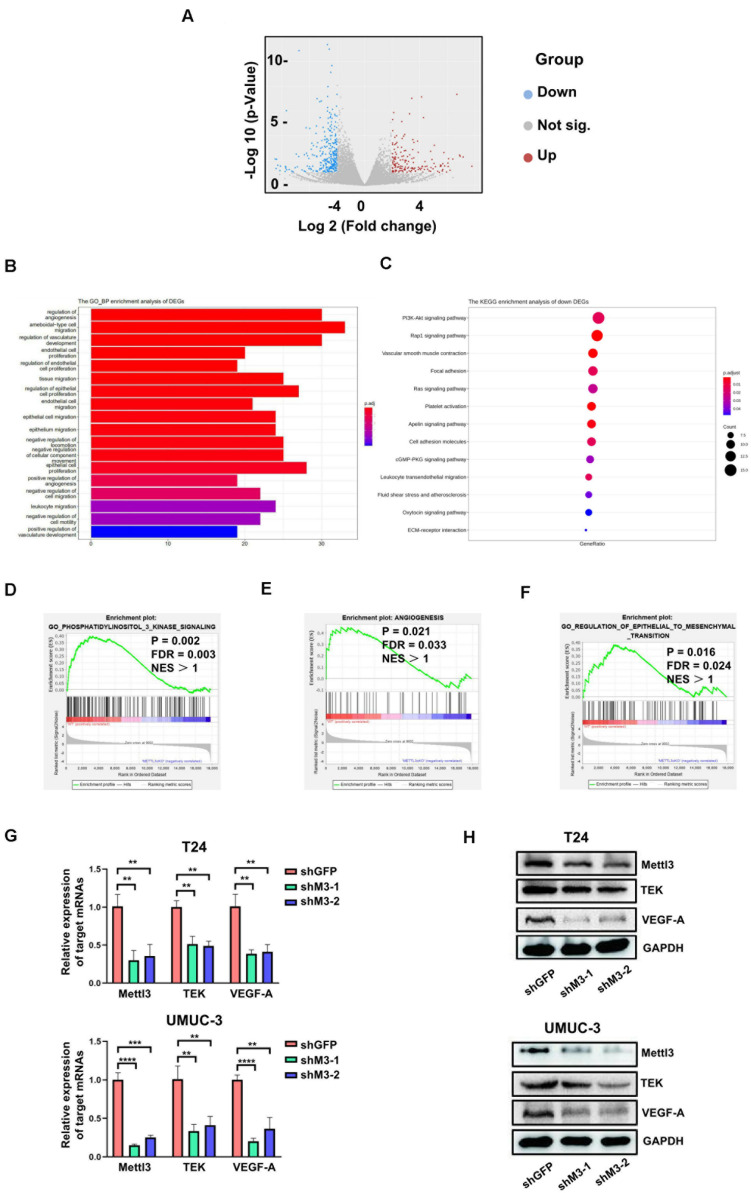
RNA-sequencing reveals Mettl3 regulates PI3K pathway and angiogenesis in bladder cancer (BCa). **(A)** The differentially expressed genes (DEGs) were shown by volcano scatterplot. Blue dots represented downregulated genes by deletion of Mettl3, gray for not significantly changed, and the red one for the upregulated genes. **(B)** The Gene Ontology Biological Process enrichment analysis of DEGs identified the most relevant function modulated by Mettl3. **(C)** The Kyoto Encyclopedia of Genes and Genomes (KEGG) enrichment analysis was conducted to examine downstream signaling pathway by monitoring down DEGs. **(D)** Gene Set Enrichment Assay plots of Gene Ontology (GO) phosphatidylinositol 3 kinase signaling for Mettl3-regulated genes from RNA-seq datasets. **(E)** Gene Set Enrichment Assay plots of angiogenesis for Mettl3-regulated genes from RNA-seq data. **(F)** Gene Set Enrichment Assay plots of GO regulation of epithelial to mesenchymal transition for Mettl3-regulated genes from RNA-seq datasets. **(G)** Quantitative PCR (qPCR) and Western blot analyses were conducted to measure the expression of AKT1 or BCL9L at messenger RNA (mRNA) and protein level in BCa cell lines. **(H)** The transcripts and proteins level of tyrosine kinase endothelial (TEK) and vascular endothelial growth factor A (VEGF-A) were examined by qPCR and Western blot experiments *in vitro*. **p < 0.01; ****p < 0.0001.

To determine the downstream mechanism of how Mettl3 regulates BCa progression, RNA-sequencing was conducted to investigate the mechanisms of Mettl3 in modulating downstream targets upon control and *Upk3a*/*K14*^*CreER*^; *Mettl3 cKO^*flox/flox*^* mice, and differentially expressed genes were shown by separating into down-, not significant (not sig.), and upregulation with the use of the volcano scatterplot (Figure 4A). Gene Ontology of analysis in DEGs associated with biological process enrichment upon control and Mettl3 KO mice revealed that those genes were selectively enriched by the regulation of angiogenesis, ameboidal-type cell migration, regulation of vasculature development, and endothelial cell proliferation, which was likely involved in construction of tumor microenvironment and sustaining tumor metastasis (Figure 4B). Next, we performed the KEGG enrichment analysis of down DEGs to systematically examine the cellular pathways affected by exhaustion of Mettl3, and the PI3K-Akt signaling pathway was discovered to be the top enrichment of 13 pathways (Figure 4C). In addition, some representative oncogenic features, such as PI3K signaling, epithelial–mesenchymal transition, and angiogenesis were statistically enriched in Mettl3 conditional knockout (KO) mice, elucidating that Mettl3 might regulate BCa in the aspect of oncogenesis, metastasis, and tumor angiogenesis-mediated chemoresistance (Figures 4D–F). To further test this hypothesis, shRNAs targeting Mettl3 were applied to inhibit Mettl3 expression *in vitro*. Consistent with the results *in vivo*, downregulation of Mettl3 in T24 or UMUC-3 cells could suppress the transcripts and proteins of TEK and VEGF-A, which were the participants of PI3K/AKT pathway (Figures 4G,H).

### Mettl3-Dependent m^6^A Modification Upon Target mRNAs Correlated With Tumor Angiogenesis and Pathways in Cancer

**FIGURE 5:**
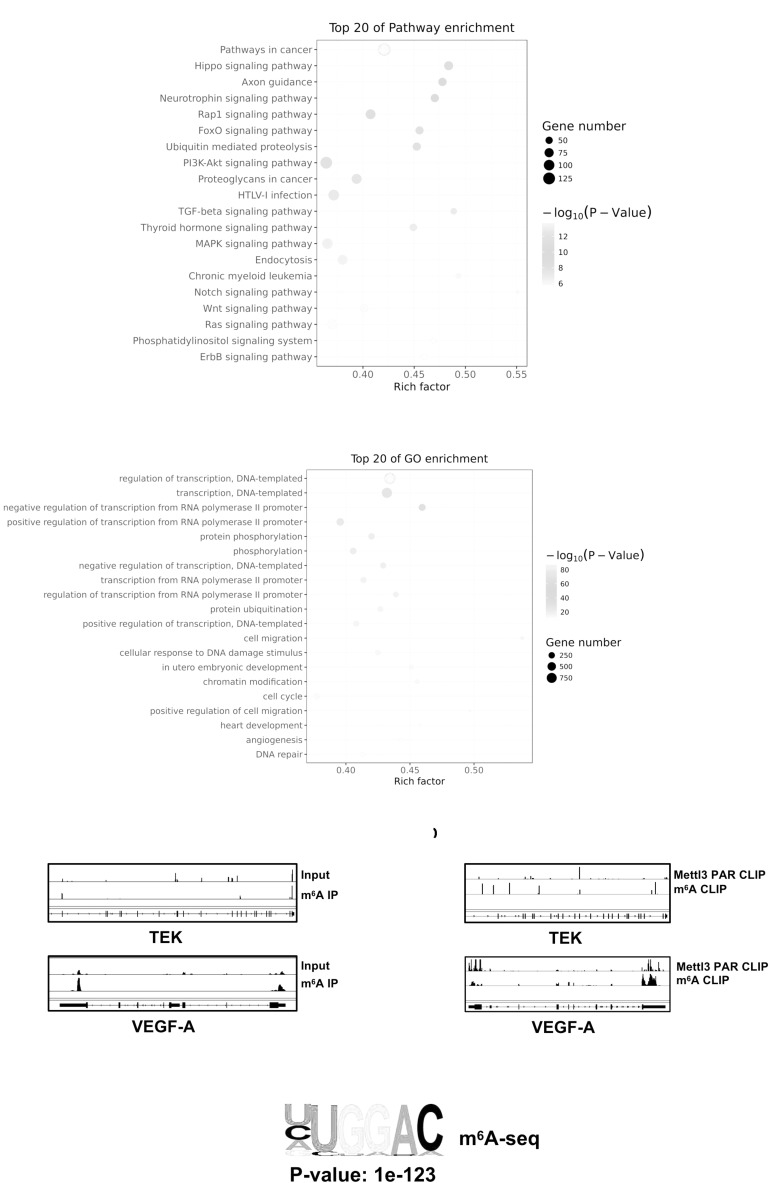
Mettl3-mediated m^6^A modification of genes correlated with PI3K pathway and angiogenesis. **(A)** Top 20 of pathway enrichment related to regulation by Mettl3 was displayed by bubble chart. **(B)** The m^6^A-enriched genes were analyzed by Gene Ontology Biological Process exhibiting top 20 Gene Ontology (GO) enrichment process. **(C)** Integrative Genomics View (IGV) tracks showing the distributions of Mettl3 binding areas and m6A reads across TEK and VEGF-A mRNAs generated from meRIP-seq. **(D)** IGV tracks showing the distributions of Mettl3 binding areas and m^6^A reads across TEK and VEGF-A mRNAs generated from Mettl3 PAR CLIP-seq or m^6^A CLIP-seq. **(E)** The most common methylated consensus motif of “GGAC” catalyzed by Mettl3 was determined by HOMER (http://homer.ucsd.edu/homer/ngs/peakMotifs.html).

To investigate whether Mettl3-mediated m^6^A modification on direct targets could effectively influence biological process of BCa, we conducted methylated meRIP-seq to identify the role of Mettl3 in BCa. First, the Pathway Analysis displaying an obviously strong enrichment for pathways in cancer proved the great potential of Mettl3 in regulating BCa progression (Figure 5A). The GO analysis on the gene set enriched by m^6^A indicated that Mettl3-medited methylation might participate in transcription regulation, protein, phosphorylation and angiogenesis (Figure 5B). We then applied Integrative Genomics View (IGV) software to search for the possible targets based on our meRIP-seq datasets and found that m^6^A peaks were abundant upon TEK and VEGF-A genes that were responsible for tumor angiogenesis (Figure 5C). Moreover, to investigate the possible m^6^A modification sites of the target genes, we analyzed online database with Mettl3 PAR CLIP and m^6^A CLIP using HeLa cell lines and identify specific modified base sites (Figure 5D) (Niu et al., 2013; Liu et al., 2014; Wang et al., 2014). Consistent with the previous reports, our results showed the most enriched motif “GGAC” in the specific regions of corresponding mRNAs (Figure 5E).

### Mettl3 Promotes Bladder Tumor Angiogenesis via Modulating TEK and VEGF-A

**FIGURE 6:**
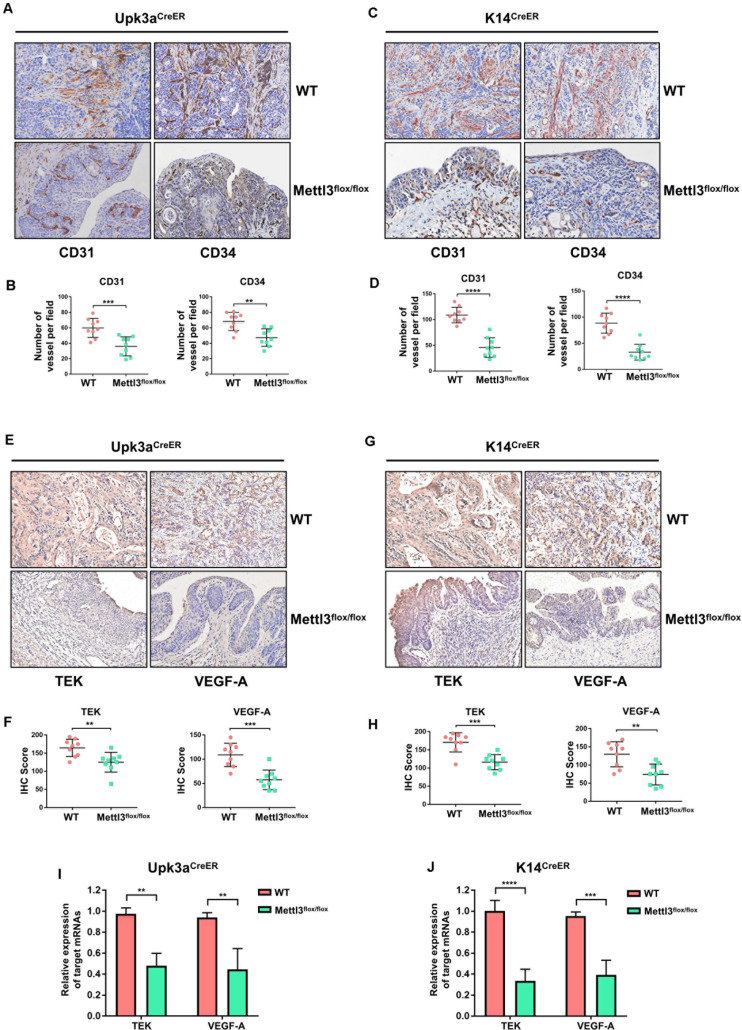
Deletion of Mettl3 repressed tyrosine kinase endothelial (TEK)/vascular endothelial growth factor A (VEGF-A)-dependent tumor angiogenesis. **(A)** CD31 and CD34 were evaluated by immunohistochemistry (IHC) staining in *Upk3a*^*CreER*^ WT (up) and *Upk3a*^*CreER*^ Mettl3 KI (bottom) mice. Scale bars, 100 μm. **(B)** The expression level of CD31 (left) and CD34 (right) was counted on the basis of their staining intensity and positive cells under a double-blind assessment. **(C)** Representative images showing IHC staining of CD31 (left) or CD34 (right) upon the sections obtained from *K14*^*CreER*^ wild-type and *K14*^*CreER*^ Mettl3 KI mice. Scale bars, 100 μm. **(D)** The variation of CD31 or CD34 caused by deletion of Mettl3 among K14-oringinated cancer cells was quantified by IHC scoring. **(E)** Representative images of IHC staining for Mettl3 targets. TEK and VEGF-A were dramatically reduced after specific ablation of Mettl3 in urothelial cells. Scale bars, 100 μm. **(F)** IHC score showing the difference of TEK and VEGF-A expression in tumor samples. **(G)** Reduction in TEK and VEGF-A expression level by loss of Mettl3 in cells from which basal layer of the epithelium originated. Scale bars, 100 μm. **(H)** IHC score was computed according to the IHC staining of *K14*^*CreER*^ mice with or without eliminating Mettl3. **(I)** Examination of TEK and VEGF-A mRNAs level from bladder tissue with or without Mettl3 knockout by the qPCR experiments. **(J)** The transcription level of TEK and VEGF-A in *K14*^*CreER*^ mice was detected by qPCR.

For comprehensive understanding of the internal regulatory mechanisms that Mettl3 mediated bladder tumor angiogenesis, we utilized immunostaining to detect and localize the angiogenesis-related factors *in vivo*. Given that CD31 and CD34 were the primary indicators of endothelial cells and vascular-associated tissue, we explored their expression pattern in BCa sections. BCa with knockout of Mettl3 in the urothelium exhibited apparent inhibition of angiogenesis (Figures 6A,B). Similarly, the angiogenesis capacity was markedly declined among *K14*^*CreER*^, *Mettl3*^*flox/flox*^ BCa tissues (Figures 6C,D).

We next quested whether the loss of Mettl3 was able to inhibit the expression of specific targets involved in tumor angiogenesis such as TEK and VEGF-A. IHC staining for assessment of downstream targets suggested that inducible knockout of Mettl3 in Upk3a-originated BCa cells significantly downregulated TEK and VEGF-A (Figures 6E,F). Coincidently, these phenomena also could be observed in tumor sections collected from *K14*^*CreER*^ WT and KO mice (Figures 6G,H). Concordant results were found performing RT-qPCR for detecting the mRNA level of TEK and CD34. Relative mRNA expression of TEK and VEGF-A among the *Upk3a*^*CreER*^, Mettl3 KO mice exerted dramatically reduced versus *Upk3a*^*CreER*^, *Mettl3*^*wt/wt*^ (Figure 6I). Furthermore, ablation of Mettl3 in K14-derived BCa cells dramatically decreased VEGF-A level (Figure 6J). Collectively, these data have testified that Mettl3 exerted its effect on tumor angiogenesis, supporting the role of Mettl3 as a collaborator of BCa progression by modulation of neovascularization.

## Discussion

Recently, RNA m^6^A methyltransferase Mettl3 has been reported to modulate AFF4/NF-κB/MYC signaling for BCa progression *in vitro* (Cheng et al., 2019). However, the role of Mettl3 in BCa progression and tumor microenvironment regulation *in vivo* remains elusive. In this study, we show that Mettl3 facilitates BCa progression by mediating tumor angiogenesis *in vivo* using transgenic mouse model. Conditional depletion of Mettl3 among the urothelium or K14^+^ CSCs significantly restricts oncogenesis and prevents BBN-induced BCa from progressing to more aggressive stage. In addition, Mettl3 ablation leads to downregulation of proliferative index protein Ki67 and upregulation of apoptosis-related Caspase-3, indicating a role of Mettl3 in cell proliferation and survival of BCa. Consistent with the data from BCa mouse model, these phenotypes were also observed *in vitro* by impairment of Mettl3, which induced the inhibition of corresponding downstream targets like AKT1 or BCL9L. The global transcriptome sequencing with KEGG analysis reveals that Mettl3 is closely related to tumor angiogenesis. Furthermore, data generated from m^6^A-squencing portray a regulatory network of Mettl3-modulated TEK/PI3K/VEGF axis, which propels the angiogenesis surrounding tumor cells. Indeed, the IHC staining of blood vessel markers such as CD31 and CD34 confirmed that the absence of Mettl3 in the urothelium or K14^+^ CSCs reduces the level of angiogenesis in BCa tissues. Our findings offer convincing evidence of Mettl3 in epigenetically modifying TEK/PI3K/VEGF cascades involving in angiogenesis, suggesting that therapeutically targeting Mettl3 may provide one of the alternatives for antiangiogenesis therapy in bladder carcinoma.

The m^6^A modification catalyzed by Mettl3 has been proven to control specific target mRNAs translation efficiency and stability, which plays a key role in promoting progression of various cancers including lung cancer, endometrial cancer, leukemia, and colorectal cancer (Barbieri et al., 2017; Choe et al., 2018; Liu et al., 2018; Li et al., 2019). Mechanically, Mettl3 could activate zinc finger MYM-type containing 1 (ZMYM1) toward genes complex of chromatin via m^6^A modification, hence promoting epithelial–mesenchymal transition (EMT) and metastasis of gastric cancer (Yue et al., 2019). Herein, we report that the mRNAs enriched by m^6^A peaks are downregulated by loss of Mettl3 likely as a result of mRNA instability. Furthermore, combining analyses of Mettl3 PAR CLIP and m^6^A CLIP sequencing, we have found possible sites bound by Mettl3 for m^6^A modification upon genes of TEK and VEGF-A. However, whether the extinction of Mettl3 interferes the translation efficiency of these targets is still unclear, which calls for further research.

Insights of regulatory mechanisms in tumor angiogenesis expand our horizons of the crosstalk between tumor cells and surrounding microenvironment. In our study, we have revealed a role of Mettl3 in angiogenesis of bladder cancer. A previous study has shown that human adipose stem cells (hASCs) can be activated by breast cancer cells, hence facilitating tumor growth and aggressiveness by promoting angiogenesis (Paino et al., 2017). However, whether Mettl3 promotes angiogenesis via interaction with preexisting vasculature endothelial cells or other mechanism, such as hASCs activation, will require further investigation. Previous study has shown Ang2-TEK signaling as a key regulator in lymphogenous metastasis (Gengenbacher et al., 2020). VEGF-A as a downstream factor of Zeb1 enhances breast cancer cells’ aggressiveness through sustaining stemness (Jiang et al., 2020). Indeed, the regulatory networks of epigenomics, angiogenesis, and stemness maintenance are intricate. A recent study describes a more faithful murine BCa model that carries *Upk3a-Cre^*ERT*2^; Trp53^*L/L*^; Pten^*L/L*^*; *Rosa26*^*LSL–Luc*^ tumor applied to excavate the biology of BCa (Saito et al., 2018). Another research indicates that the subpopulation of bladder basal cells characterized by K14 possesses self-renewal ability, and these cells are defined as the origin of urothelial cancer, which contributes to BCa progression (Papafotiou et al., 2016). Encouragingly, a combination of these rationales of signal networks in BCa may depict the extensive interactions of stemness maintenance, epigenetic modulation, and tumor angiogenesis. Our results have revealed a novel mechanism of Mettl3 by which it mediates m^6^A modulation of TEK and VEGF-A to promote tumor angiogenesis.

In summary, our data demonstrate the critical role of Mettl3 in driving BCa progression, as featured by expediting angiogenesis surrounding tumor cells, and unveil a previously unrecognized signaling axis involving Mettl3-TEK-VEGF-A-CD31/CD34 in bladder malignancy. More importantly, this study proposes that targeting Mettl3 might bring profound influence on conquering chemotherapy resistance and immunotherapy tolerance caused by tumor angiogenesis, offering therapeutic alternatives to patients suffering this treatment dilemma.

## Data Availability Statement

The raw data supporting the conclusions of this article will be made available by the authors, without undue reservation.

## Ethics Statement

All institutional and national guidelines for the care and use of laboratory animals were followed.

## Author Contributions

DC and LP conceived and were in charge of this project. GW, YD, KL, MC, GX, XW, SC, ZC, JC, and XX conducted the experiments under the supervision of DC. KL and R-sL performed bioinformatics analysis under the supervision of DC and LP. GW and DC wrote the manuscript. All authors contributed to the article and approved the submitted version.

## Conflict of Interest

The authors declare that the research was conducted in the absence of any commercial or financial relationships that could be construed as a potential conflict of interest.

## Footnotes

1 https://toppgene.cchmc.org/enrichment.jsp

2 https://www.kegg.jp/kegg/pathway.html

3 https://www.gsea-msigdb.org/gsea/downloads.jsp
