# Corrigendum: Deficiency of Mettl3 in Bladder Cancer Stem Cells Inhibits Bladder Cancer Progression and Angiogenesis

**DOI:** 10.3389/fcell.2021.675417

**Published:** 2021-03-24

**Authors:** Ganping Wang, Yarong Dai, Kang Li, Maosheng Cheng, Gan Xiong, Xiaochen Wang, Shuang Chen, Zhi Chen, Jianwen Chen, Xiuyun Xu, Rong-song Ling, Liang Peng, Demeng Chen

**Affiliations:** ^1^Center for Translational Medicine, Institute of Precision Medicine, The First Affiliated Hospital, Sun Yat-sen University, Guangzhou, China; ^2^Institute for Advanced Study, Shenzhen University, Shenzhen, China; ^3^Department of Oral and Maxillofacial Surgery, Hospital of Stomatology, Guanghua School of Stomatology, Sun Yat-sen University, Guangzhou, China; ^4^Department of Oncology, Chinese PLA General Hospital, Beijing, China

**Keywords:** Mettl3, cancer stem cell, bladder cancer, m^6^A, angiogenesis

There is an error in the Funding statement. The correct number for Natural Science Foundation of Guangdong Province is no. 2018A030313610, and the correct number for National Natural Science Foundation of China is no. 81872409.

The authors apologize for this error and state that this does not change the scientific conclusions of the article in any way. The original article has been updated.

